# A high-throughput phenotypic screen identifies clofazimine as a potential treatment for cryptosporidiosis

**DOI:** 10.1371/journal.pntd.0005373

**Published:** 2017-02-03

**Authors:** Melissa S. Love, Federico C. Beasley, Rajiv S. Jumani, Timothy M. Wright, Arnab K. Chatterjee, Christopher D. Huston, Peter G. Schultz, Case W. McNamara

**Affiliations:** 1 California Institute for Biomedical Research, La Jolla, California, United States of America; 2 Department of Medicine, University of Vermont College of Medicine, Burlington, Vermont, United States of America; 3 Department of Chemistry and The Skaggs Institute for Chemical Biology, The Scripps Research Institute, La Jolla, California, United States of America; McGill University, CANADA

## Abstract

Cryptosporidiosis has emerged as a leading cause of non-viral diarrhea in children under five years of age in the developing world, yet the current standard of care to treat *Cryptosporidium* infections, nitazoxanide, demonstrates limited and immune-dependent efficacy. Given the lack of treatments with universal efficacy, drug discovery efforts against cryptosporidiosis are necessary to find therapeutics more efficacious than the standard of care. To date, cryptosporidiosis drug discovery efforts have been limited to a few targeted mechanisms in the parasite and whole cell phenotypic screens against small, focused collections of compounds. Using a previous screen as a basis, we initiated the largest known drug discovery effort to identify novel anticryptosporidial agents. A high-content imaging assay for inhibitors of *Cryptosporidium parvum* proliferation within a human intestinal epithelial cell line was miniaturized and automated to enable high-throughput phenotypic screening against a large, diverse library of small molecules. A screen of 78,942 compounds identified 12 anticryptosporidial hits with sub-micromolar activity, including clofazimine, an FDA-approved drug for the treatment of leprosy, which demonstrated potent and selective in vitro activity (EC_50_ = 15 nM) against *C*. *parvum*. Clofazimine also displayed activity against *C*. *hominis*–the other most clinically-relevant species of *Cryptosporidium*. Importantly, clofazimine is known to accumulate within epithelial cells of the small intestine, the primary site of *Cryptosporidium* infection. In a mouse model of acute cryptosporidiosis, a once daily dosage regimen for three consecutive days or a single high dose resulted in reduction of oocyst shedding below the limit detectable by flow cytometry. Recently, a target product profile (TPP) for an anticryptosporidial compound was proposed by Huston et al. and highlights the need for a short dosing regimen (< 7 days) and formulations for children < 2 years. Clofazimine has a long history of use and has demonstrated a good safety profile for a disease that requires chronic dosing for a period of time ranging 3–36 months. These results, taken with clofazimine’s status as an FDA-approved drug with over four decades of use for the treatment of leprosy, support the continued investigation of clofazimine both as a new chemical tool for understanding cryptosporidium biology and a potential new treatment of cryptosporidiosis.

## Author summary

Diarrheal diseases cause significant morbidity and mortality in the developing world. A recent multi-site study investigating diarrheal disease identified *Cryptosporidium* as one of the leading causes, responsible for upwards of 25% of cases in some regions. Currently approved therapy for cryptosporidiosis is limited to nitazoxanide, which is approved only for children ages 1–11 years. Nitazoxanide demonstrates moderate efficacy in immunocompetent patients and poor efficacy in immunodeficient patients. The limited treatment options underscore an opportunity to reduce the global burden of diarrheal disease by developing novel medicines with increased efficacy against *Cryptosporidium* spp. We report the largest high-throughput screen for anticryptosporidial compounds to date, leading to the identification of the FDA-approved drug clofazimine as a potential new treatment for cryptosporidiosis, and as a tool to probe the biology of this pathogen.

## Introduction

*Cryptosporidium* species are apicomplexan protozoans that are important causes of diarrhea in humans and some domestic animals. The parasite relies on an oral-fecal route of transmission, and ingestion of water or food contaminated with *Cryptosporidium* oocysts may lead to infection. Upon ingestion, oocysts are activated, releasing four sporozoites which then invade host epithelial cells in the small intestine [1]. In some severe cases, infection may expand beyond the gastrointestinal tract and into the respiratory tract—a complication most often seen in patients with human immunodeficiency virus (HIV) [2–4]. The life cycle is not well understood, although *Cryptosporidium* undergoes both asexual and sexual replication within a single host, ultimately leading to the generation of environmentally-hardy infectious thick-walled oocysts that are excreted with the host feces [5]. Acute and persistent watery diarrhea with concomitant oocyst shedding are hallmarks of cryptosporidiosis [6]. Clinically, the two most relevant species that cause human cryptosporidiosis are *C*. *hominis* and *C*. *parvum*, and distribution of each species varies greatly depending on the region and geography. A large molecular epidemiology study carried out by the Global Enteric Multi-Center Study (GEMS) revealed *Cryptosporidium* spp. to be the second-leading cause of life-threatening diarrheal disease in young children [7]. A second study by MAL-ED confirmed the significant contribution of *Cryptosporidium* to diarrheal disease burden in infants 12 months and younger [8]. While rotavirus continues to be the most common cause of severe pediatric diarrheal disease, 8–30.5% of cases, dependent upon location and age range, are now attributed to cryptosporidiosis [9–13].

In general, severe infectious diarrheal diseases cause dehydration and malnutrition due to low retention of nutrients [14]. Young children are particularly vulnerable to the untoward effects of severe diarrhea, which can result in death or stunted development [8, 15]. Currently, there is only one approved drug to treat *Cryptosporidium* infections—nitazoxanide [16]. The efficacy of nitazoxanide has been questioned [17] and appears to be dependent upon a competent immune system. This is notable because young children and immunodeficient individuals are disproportionally affected by cryptosporidiosis [18], and nitazoxanide demonstrates very poor efficacy in AIDS patients [19], highlighting an urgent unmet medical need among this comorbid patient population.

Drug discovery efforts against cryptosporidiosis have been limited to a few targeted mechanisms in the parasite and whole cell phenotypic screens against small, focused collections of compounds [20–22]. The most advanced compound from these efforts, bumped kinase inhibitor 1294 (BKI-1294), is a putative inhibitor of *C*. *parvum* calcium-dependent protein kinase 1 (CDPK1) [23] which is a validated target in other protozoans, including *Toxoplasma gondii* [24] and *Plasmodium* spp. [25]. BKI-1294 has demonstrated efficacy in a mouse model of chronic cryptosporidiosis when dosed once per day for ten consecutive days at 100 mg/kg [23]. Active compounds have also emerged from screens for inhibitors of inosine-5’-monophosphate dehydrogenase [26], and polyamine analogues [27]. Despite these efforts there remains a dearth of compounds in the drug discovery pipeline, a limited biological understanding, and a lack of available tools to study this parasite. Therefore, we undertook a high-throughput screen to identify compounds that may lead to effective new treatments for cryptosporidiosis.

## Results

### A phenotypic assay for high-throughput screening

**Fig 1:**
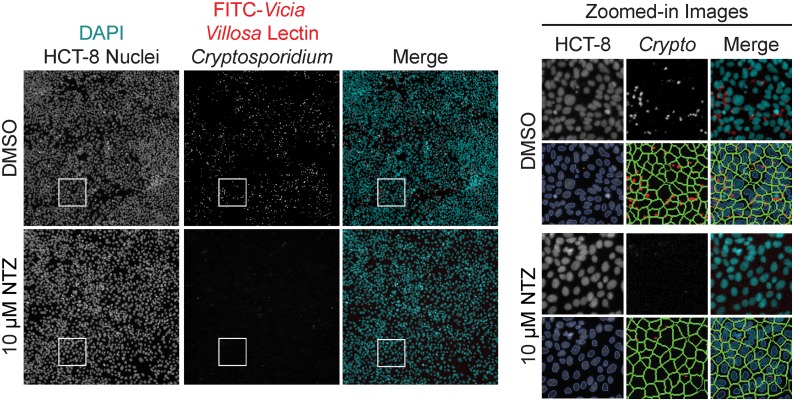
Screening images and software analysis. Representative images and software analysis from 1536-well High Content Imaging on Cellomics CellInsight CX5. (Left) Images of two wells either mock-treated with dimethyl sulfoxide (DMSO) or 10 μM nitazoxanide (NTZ). A merged image of the two fluorescent channels are artificially colored to show contrast: DAPI in cyan and FITC in red. Well area for each full-sized image is 802,511.39 μm^2^. (Right) Zoomed-in images from the inset white squares in the left panel (area is 24,318.53 μm^2^) are shown. The image overlays are applied by the imaging software to assess the assay metrics: HCT-8 cell count and *Cryptosporidium* spot count. First, the host cell nuclei are identified and counted based on DAPI signal; cell debris and other particles are rejected based on a size filter (orange). Next, a region of interest, or “cell area” is drawn around each host cell nuclei to encompass where *Cryptosporidium* parasites may be located. Finally, the software identifies and counts “spots” within the “cell area” based on signal from the FITC-conjugated *Vicia villosa* lectin (red).

**Fig 2:**
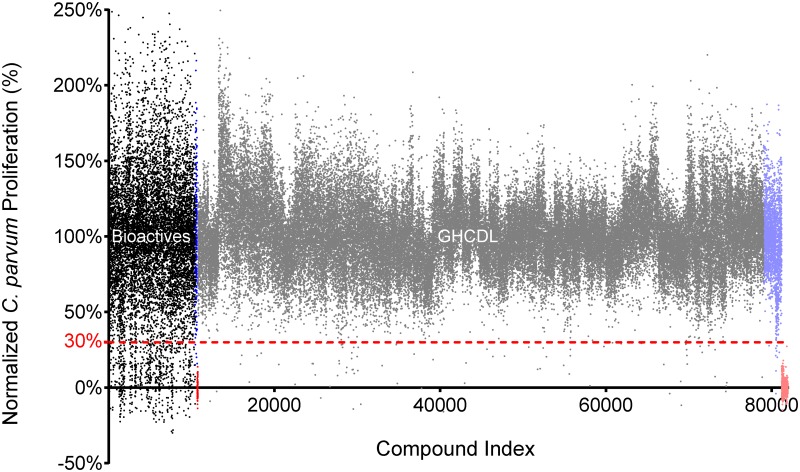
High-throughput screening results of 78,942 compounds shows inherent variability. Scatter plot of normalized activity all compounds screened at 1.88 μM (Black = Bioactives; Gray = GHCDL). A strict cut-off of 70% inhibition of *C*. *parvum* proliferation (dotted red line) was applied to yield 812 primary hits. Neutral controls (0.125% DMSO; Blue = Bioactives; Lavender = GHCDL) and inhibitor controls (0.5 μM FDU; Red = Bioactives; Light red = GHCDL) are also shown to demonstrate the inherent variability in the assay.

As a starting point, we developed a whole-cell phenotypic screening platform for testing a library of small molecules. A high-content screen of *C*. *parvum*-infected human intestinal epithelial HCT-8 cells was previously developed in a 384-well format by Bessoff et al. [20] to measure *C*. *parvum* proliferation over a 48-h period. We modified this assay for automated dispensing and further miniaturization to 1536-well format, both critical factors to support a high-throughput screening campaign (Fig 1). A small pilot screen was initiated and assay quality was determined by calculating the Z’ value. Despite miniaturization, the 1536-well assay had comparable Z’ values (0.2–0.5; S1 Table) to the published 384-well assay format when comparing control wells containing DMSO to wells treated with the positive controls nitazoxanide (NTZ) and 5-fluoro-2’deoxyuridine (FDU). Like the 384-well assay, a strict hit cut-off for parasite proliferation was applied to avoid high false-positive hit rates created by higher than average well-to-well variability (Fig 2). Initial pilot screens with a final compound concentration of 1.88 μM confirmed that typical hit reconfirmation rates of 40–50% were observed when applying a 70% inhibition cut-off. Because a significant decrease in hit reconfirmation, reflecting a significantly higher false-positive rate, was observed when the hit cut-off was reduced, the 70% inhibition cut-off was applied for all primary screens.

The miniaturized, fully automated design of the *C*. *parvum* proliferation assay enables a screen throughput of approximately 40,000 compounds per assay. We screened two modestly sized compound libraries to validate this assay and identify new anticryptosporidial compounds: a Bioactive (10,000) set assembled by the California Institute for Biomedical Research (Calibr), and the Global Health Chemical Diversity Library (GHCDL; 69,000) provided by the University of Dundee Drug Discovery Unit. A primary screen of these libraries revealed highly divergent hit rates that varied by 70-fold and yielded 106 reconfirmed hits (75 from the Bioactive set; 31 from the GHCDL; Table 1). These hits were resupplied from high-purity powder stocks and assayed in dose-response. Compounds with a half maximal effective concentration (EC_50_) against intracellular *C*. *parvum* of less than 1 μM (49 from Bioactives and 18 from GHCDL) and no discernable HCT-8 cytotoxicity (≥ 10-fold the observed EC_50_ value) were deemed selective, potent hits (10 from Bioactives and 2 from GHCDL; S2 Table). The high attrition of screen hits at this stage was directly attributable to false positives from compounds that demonstrated poor selectivity and were generally cytotoxic to the host HCT-8 line and/or against additional counter-screened mammalian cell lines (HepG2 and HEK293T). The remaining selective compounds were advanced for hit validation against *Cryptosporidium hominis*.

**Table 1:** Screening results, including compound totals for each library.

	Bioactives	GHCDL
Total compounds	10,453	68,489
Hits (≥ 70% inhibition)	734	78
Raw hit rate (%)	7.02	0.11
Cytotoxicity filter	130	N/A
Filtered hit rate (%)	1.24	0.11
Reconfirmed hits	75	31
Reconfirmation rate (%)	57.7	39.7
EC_50_ ≤ 1 μM	49	18
Selected filtered hits (CC_50_:EC_50_ ≥ 10)	10	2

A host cell cytotoxicity filter was applied (≥ 40% HCT-8 cell inhibition) and the remaining compounds were tested in triplicate for reconfirmation. Reconfirmed hits were then tested in dose-response, and compounds were further filtered by potency and selectivity (final row).

### Anticryptosporidial activity of screening hits confirmed against *C*. *hominis*

**Fig 3:**
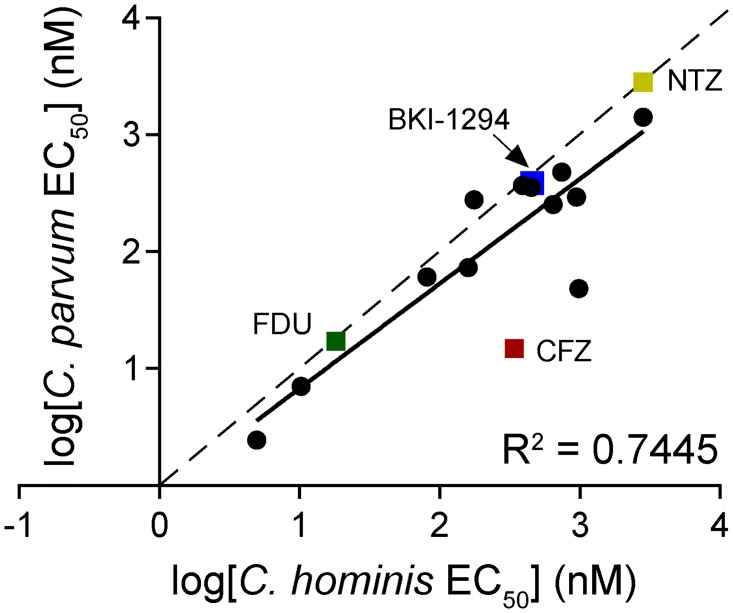
Screening hits show activity against two species of *Cryptosporidium*. Twelve filtered hits and multiple controls were tested against *C*. *parvum* and *C*. *hominis* for cross-species confirmation. Data points are log-transformed EC_50_ values. Controls and compounds of interest are denoted with a square symbols and labeled. The correlation is shown by the trendline slope (solid line, m = 0.7445) compared to a perfect linear correlation (dotted line, m = 1).

Methods for continuous, in vitro cultivation of *Cryptosporidium* spp. have not been well established. Instead, in vivo oocyst propagation of *C*. *parvum* is performed in calves, a more promiscuous *Cryptosporidium* species for animal and human infections. For *C*. *hominis*, gnotobiotic piglets are the primary means of propagation. The calf is the more accessible model, making *C*. *parvum* the more convenient species to screen. However, *C*. *hominis* is equally clinically relevant with regards to human cryptosporidiosis. Thus, compounds identified against *C*. *parvum* were counter-screened against *C*. *hominis* to confirm that these compounds were active against both species. Given that the genomes of these two species exhibit extremely high synteny and sequence identity (95–97%) [28], compound potency was expected to also be highly correlated. High purity powders of the 10 filtered *C*. *parvum* Bioactives screen hits and both GHCDL screen hits were tested against *C*. *hominis* (TU502) to confirm activity against both species. Surprisingly, the species-specific activities the screening hits and control compounds, NTZ and FDU, were determined to be only modestly correlated (R^2^ = 0.7445; Fig 3). While no compounds demonstrated species-exclusive activity, there were notable outliers with differential activities between species, including the known compounds clofazimine and cyclosporine (~20-fold more potent against *C*. *parvum*).

### Clofazimine, a potent inhibitor of *Cryptosporidium* asexual development

**Fig 4:**
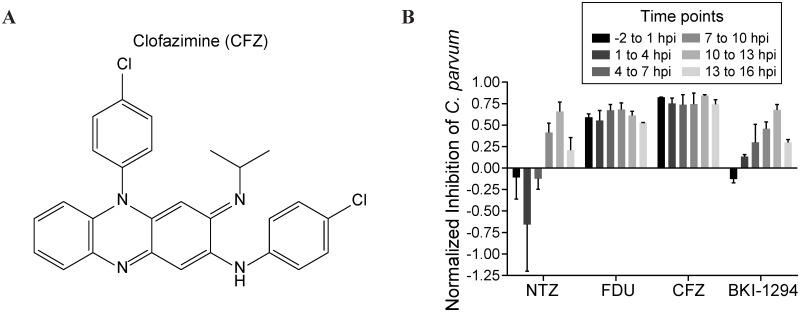
In vitro characterization of CFZ. (A) Chemical structure of CFZ. (B) CFZ inhibited *C*. *parvum* proliferation by ≥ 70% at every point of the asexual life cycle. The first asexual life cycle after infection was evenly divided into six 3-h blocks, and labeled as hours post infection (hpi). Infected cells were treated by one of four compounds at the EC_99_ for 3 h followed by drug washout, and then allowed to continue growing until 48 hpi, when they were fixed, stained, imaged, and analyzed for *C*. *parvum* proliferation. EC_99_ values: NTZ = 8 μM; FDU = 100 nM; CFZ = 30 nM; BKI-1294 = 2 μM. Data shown are the mean ± SEM of two independent experiments.

Validated compounds that had the greatest potency were all derived from the Bioactive compound collection comprised of known drugs and annotated compounds. The five most potent compounds against *C*. *parvum* were Gö6976 (EC_50_ = 2.5 nM; S1 Fig), monensin (EC_50_ = 7 nM), clofazimine (EC_50_ = 15 nM; Fig 4A), cyclosporine (EC_50_ = 48 nM), and MST-312 (EC_50_ = 61 nM). Three of these compounds were designed to target mammalian enzymes: Gö6976 is a potent inhibitor of protein kinase C (isotypes α and β) and of the tyrosine kinases JAK 2 and FLT3 [29, 30], whereas MST-312, also known as Telomerase Inhibitor IX, is a potent, reversible inhibitor of telomerase activity and arrests cells in the G0-G1 phase during the cell cycle [31]. Finally, cyclosporine is an inhibitor of the phosphatase activity of calcineurin with potent immunosuppressive properties that is indicated for graft-versus-host disease, rheumatoid arthritis, psoriasis, and other immune-related diseases [32]. The other two compounds, monensin and clofazimine (CFZ), are both antibiotics. Monensin is an ionophore antibiotic commonly added to cattle feed [33, 34] and CFZ is an FDA-approved drug for the treatment of leprosy, a disease caused by chronic infection of the bacteria *Mycobacterium leprae* and *Mycobacterium lepromatosis*. The inherent limitation of phenotypic screens prevents elucidation of whether the inhibition of *C*. *parvum* proliferation is attributable to modulation of the human host cell or to direct inhibition of a *Cryptosporidium* target. Regardless, Gö6976, MST-312, cyclosporine, and monensin all demonstrated a limited therapeutic index when activity against *C*. *parvum* was compared to toxicity for mammalian cell lines, or had undesirable effects, and these compounds were therefore deprioritized. The remaining compound, CFZ, is of particular interest based on a very good safety profile in humans with dosage regimens lasting up to three years [35].

We further characterized CFZ in an assay evaluating the cidal activity throughout the parasite lifecycle. We examined short, three-hour compound treatments of in vitro cultures, followed by compound washout, that covered the complete asexual life cycle, believed to be 13–15 h post invasion [36]. The first treatment group had compound added to the cell monolayers 2 h prior to infection with *C*. *parvum* sporozoites from excysted oocysts. The treatment interval lasted 3 h before washout with fresh medium; infected cells were allowed to grow until 48 h post infection before fixation. We treated assay plates with NTZ, FDU, BKI-1294, and CFZ at their EC_50_, EC_99_, or 3×EC_99_ value at the designated intervals throughout the lifecycle (EC_99_ data shown; Fig 4B). Strikingly, CFZ inhibited parasite growth >75% at the EC_99_ at every time point, indicating that the effect of CFZ is irreversible within 3 h of treatment at any point throughout the parasite’s asexual life cycle. FDU, a cytotoxic pyrimidine analog, also showed strong inhibition of growth across all time points. Though FDU is generally cytotoxic, it has been previously shown to be a substrate of *C*. *parvum* thymidine kinase and inhibit *C*. *parvum* growth without affecting HCT-8 host cells [37]. Interestingly, both nitazoxanide and BKI-1294 showed the greatest effect on parasite growth at the end of the life cycle (10–13 h post infection), and at all other time points showed <60% inhibition, even when concentrations were increased to 3×EC_99_ (S2 Fig). As CFZ is very lipophilic, we sought to determine whether intracellular accumulation was affecting the strong inhibition across each time point. Upon pre-treatment with each compound in a dose-response titration, we observed that both FDU and CFZ were retained by host cells and exhibited near equipotent inhibition of *C*. *parvum* proliferation despite compound removal from the assay media by repeated washes (S3 Table). This retention effect likely contributes to the high inhibition of *C*. *parvum* proliferation observed at each time point in the washout assay. However, high retention, and subsequent high activity against parasite proliferation, may be a desirable property to promote high in vivo efficacy. The excellent activity profile of CFZ compared to BKI-1294 supported in vivo pharmacokinetic and efficacy studies in a mouse model of cryptosporidiosis.

### In vivo pharmacokinetics and efficacy of clofazimine

**Fig 5:**
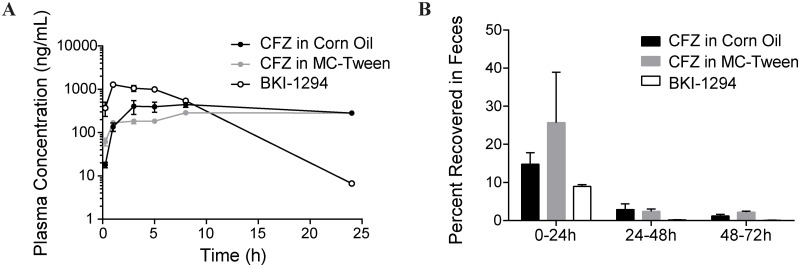
Pharmacokinetic properties of CFZ. (A) Plasma concentration of CFZ or BKI-1294 in mice dosed with 20 mg/kg compound. CFZ was formulated in either corn oil (black) or MC-Tween (gray); BKI-1294 was formulated in 7% Tween 80, 3% ethanol, and 90% water (white). Data shown are mean ± SEM (n = 3). (B) Unchanged CFZ or BKI-1294 recovered in the feces of mice dosed in (A). Recovery was measured each day for three days. Data shown are mean ± SEM (n = 3).

CFZ has been reported to have two remarkable properties: a long drug half-life (~70 days) [38] and very high tissue distribution. This leads to a notable accumulation of CFZ throughout the body [39], including the skin, producing a strong but reversible pigmentation with chronic administration. In anticipation of advancing CFZ to a mouse model of cryptosporidiosis to determine its efficacy, we characterized the pharmacokinetic properties of this compound solubilized in corn oil or prepared as a suspension in methylcellulose and Tween 80 (MC-Tween). Healthy CD-1 mice dosed with 20 mg/kg CFZ (4 mg/mL) prepared in either formulation demonstrated comparable pharmacokinetic profiles. Plasma concentrations of CFZ peaked at 445 ± 61 ng/mL (corn oil) and 288 ± 2 ng/mL (MC-Tween) within the first 24 h after oral administration (Fig 5A). The extended biological half-life was evident as plasma concentrations remained stable for at least 20 hours after the initial maximal concentration. Mice dosed with an equivalent amount of BKI-1294 reached a higher peak (1282 ± 154 ng/mL), though the plasma concentration rapidly dropped to 6.7 ± 0.3 ng/mL within the first 24 h post dosing. Pharmacokinetic analysis was further extended to a three-day evaluation of CFZ and BKI-1294 elimination in the feces and urine of mice following a single oral dose. Within the first 24 h after oral administration, the mean (± SEM) fecal excretion was 14.8% ± 3.1% for CFZ in corn oil and 25.7% ± 13.3% for CFZ in MC-Tween, while BKI-1294 was only excreted unchanged at 8.9% ± 0.5% (Fig 5B). Continued evaluation of feces for the next 48 h showed mean excretion values < 2% for either formulation of CFZ. Parallel collection and analysis of urine over the first 48 h period revealed < 0.15% of the CFZ dosage versus 1.6% of BKI-1294 excreted in the urine (S3 Fig). In the absence of a clearly defined pharmacokinetic profile for anticryptosporidial compounds, we surmise that the detectable presence of CFZ in the feces confirms CFZ exposure throughout the entire gastrointestinal tract.

**Fig 6:**
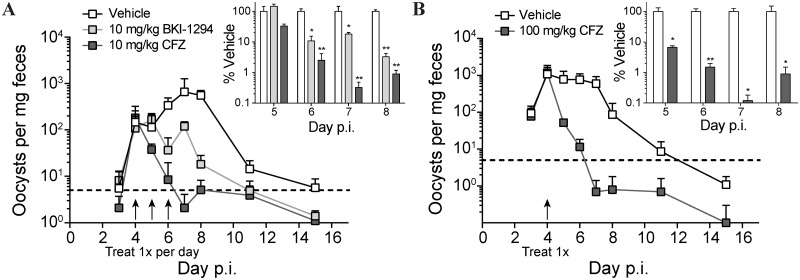
CFZ is efficacious in a mouse model of acute cryptosporidiosis. Fecal oocyst recovery from IFN-γ^-/-^ mice commenced three days after oral delivery of *C*. *parvum* oocysts. Line graph data are weight-adjusted mean oocyst counts ± SD (n = 4); inset bar graphs are mean % recovery relative to mock-treated control mice ± SEM (n = 4). (A) Mice were infected with 10^4^ oocysts then treated orally with 10 mg/kg BKI-1294 (light gray) or CFZ (dark gray) on days 4, 5, and 6 p.i. Dotted line is the reliable limit of detection. A two-way ANOVA was conducted to determine significance between mice treated with compound vs mice treated with vehicle: * p < 0.05; ** p < 0.01. (B) Mice were infected with 10^6^ oocysts then treated orally with a single dose of 100 mg/kg CFZ on day 4. Multiple Student’s t-tests were used to determine significance between vehicle-treated and CFZ-treated mice: * p < 0.05; ** p < 0.01.

The juvenile IFNγ^-/-^ mouse is susceptible to *C*. *parvum* infection via the oral route [40]. Despite the previously reported lethality, in our hands, *C*. *parvum*-infected IFNγ^-/-^ mice remained overtly asymptomatic, with infection marked by an acute period of intense oocyst shedding (3- to 8-days post infection (p.i.); reproducibly peaking at approximately one thousand oocysts per milligram of feces). This reproducible fecal oocyst shedding was measured by flow cytometry to quantify therapeutic efficacy of CFZ benchmarked against the recently published anticryptosporidial compound BKI-1294 (Fig 6A). Three daily oral doses of 10 mg/kg were given over a three-day period beginning at the onset of high-level oocyst shedding (day 4 p.i.). Within 24 h of the end of treatment (day 7), BKI-1294 had reduced oocyst shedding to 18.1% ± 2.2% of mock-dosed mice, while CFZ treatment reduced shedding to below 1% of the control group and below the reliable limit of detection (Fig 6A, inset). Rapid efficacy of CFZ was evident. Within two days of the treatment onset, shedding was reduced to 2.5% ± 1.7% of the control group, suggesting that single-dose treatment might be efficacious. To evaluate the potential for single-dose efficacy, mice were orally gavaged with an augmented infective titer of *C*. *parvum* (1×10^6^ oocysts) and treated with one 100 mg/kg oral dose of CFZ on day 4 (Fig 6B). A rapid reduction in oocyst shedding was observed for the CFZ group, with counts decreasing to the reliable limit of detection by the second day after treatment (1.5% ± 0.4% of mock-treated mice; Fig 6B, inset). In contrast, the control group of mice continued shedding close to 1×10^3^ oocysts per milligram of feces with the gradual onset of self-resolution not apparent until day 8 p.i. (4 days post treatment).

## Discussion

Phenotypic screens have been used extensively for neglected tropical diseases to spearhead drug discovery efforts and generate tool compounds for chemical biology. Here we describe the automation and miniaturization of a previously established high-content imaging phenotypic assay to identify 12 anticryptosporidial compounds. The ultimate objective of this work is to discover novel leads for the treatment of cryptosporidiosis and to identify diverse small molecules with anticryptosporidial activity that may become valuable chemical probes to study *Cryptosporidium* biology. The most promising hit compound, clofazimine (CFZ), is an FDA-approved riminophenazine antibiotic used for the treatment of leprosy that is recognized by the World Health Organization as an essential medicine. The discovery of CFZ as an anticryptosporidial compound, and the subsequent demonstration of efficacy in a mouse model of acute cryptosporidiosis has provided a novel chemical tool to help study and define the pharmacokinetic and pharmacodynamic properties that drive in vivo efficacy, and a potential drug repurposing candidate for the treatment of human cryptosporidiosis.

Aside from CFZ, the ability to prioritize anticryptosporidial screening hit compounds currently presents a challenge because a consensus profile of predictive in vitro ADME and in vivo pharmacokinetic characteristics has yet to be established. Recent insights from Gorla et al. [26] showed that in vivo efficacy of *C*. *parvum* inosine-5’-monophosphate dehydrogenase (*Cp*IMPDH) inhibitors correlated well with the compounds’ uptake and intracellular accumulation in Caco-2 cells, an established model for human intestinal epithelial cells. Conversely, high Caco-2 permeability and systemic exposure in blood plasma within this series had no correlation to efficacy in a mouse model of acute cryptosporidiosis. While these correlations are rational given the intestinal pathogenesis of this parasite, validation for whether intestinal bioaccumulation is a general characteristic of efficacious anticryptosporidial compounds or a specific driver of efficacy for the *Cp*IMPDH inhibitor series evaluated by Gorla et al. remains to be determined. To this end, the in vitro and in vivo pharmacokinetic profiles of CFZ become of considerable interest.

CFZ is known to have limited oral bioavailability, requiring it to be encapsulated as a micronized suspension in a lipid-wax base (Lamprene^®^; Novartis) to promote higher absorption in the treatment of the *Mycobacterium* spp. responsible for leprosy. Its non-optimal systemic exposure has been particularly restrictive for the development of CFZ in the treatment of *Mycobacterium tuberculosis*, the causative agent of tuberculosis [41]. While compounds for the treatment of tuberculosis require high systemic exposure, this same pharmacokinetic property may not be a limitation for the treatment of *Cryptosporidium* infections, which primarily affect the epithelium of the small intestine. The intestinal permeability and high lipophilicity of CFZ ensures retention and exposure throughout the gastrointestinal tract, reportedly leading to the highest deposition of CFZ in the jejunum and ileum [39]. Furthermore, the high bioaccumulation in the small intestine and limited compound solubility can lead to crystalline-like CFZ deposits within the cytoplasm of macrophages [42] and intestinal epithelial cells. These intestinal epithelial crystalline-like CFZ deposits are observed with as few as five doses of 200 mg/kg administered over a two-week period in a mouse model [43]. Chronic dosing (8 weeks at ~10 mg/kg/day in mice) has also been shown to lead to CFZ distribution and bioaccumulation in the mesenteric lymph nodes, spleen and fatty tissue [39]. Whereas the selective partitioning of CFZ into specific tissues may be limiting for the treatment of systemic disease, these properties appear to be favorable for efficacy against *C*. *parvum* in a mouse model of acute cryptosporidiosis.

We have not as yet established whether CFZ directly targets a parasitic pathway or modulates a host-mediated pathway essential to parasite proliferation. However, the differential in vitro activity between *C*. *parvum* (EC_50_ = 15 nM) and *C*. *hominis* (EC_50_ = 340 nM) assayed in the same in vitro host cell line are suggestive of a parasite-directed mechanism. Interestingly, CFZ has been noted to have both antibiotic and anti-inflammatory effects, and perhaps not surprisingly, it does not have a clearly defined mechanism of action (MOA) associated with either activity. CFZ has been implicated in the disruption of the K_v_1.3 potassium channel, suggesting a potential mechanism by which CFZ selectively modulates immunosuppression involving K_v_1.3-expressing T cells [44]. Mechanistic studies in mycobacterium suggest CFZ may directly compete with menaquinone, an essential cofactor of the electron transfer chain [45]. Interestingly, *Cryptosporidium* has a unique, atypical mitochondrial assembly and genes encoding proteins for electron transport or oxidative phosphorylation have yet to be identified [46]. Previously no known homologs to human potassium voltage-gated (K_v_) channels have been identified in the relevant *Cryptosporidium* spp. [47], thus confounding the mechanism by which CFZ may inhibit parasite proliferation.

Regardless of a defined MOA, its status as an FDA-approved drug and over four decades of use for the treatment of leprosy warrants further investigation of CFZ as a possible repurposing candidate for treatment of cryptosporidiosis. Recently, a target product profile (TPP) for an anticryptosporidial compound was proposed by Huston et al. [48] and highlights the need for a short dosing regimen (< 7 days) and formulations for young children (0–24 months). CFZ has a long history of use and has demonstrated a good safety profile for a disease that requires chronic dosing for a period of time ranging 3–36 months. The safety profile for young children and infants is not yet well defined and will require additional investigation. However, the targeted dosing regimen of < 7 days is a fraction of the minimal time frame for which this drug is administered to leprosy patients, and the once-daily oral administration of 10 mg/kg CFZ for three days in a mouse model of cryptosporidiosis is likely to translate favorably to human treatment. Importantly, it may be possible that all three doses could be delivered within a single 12 to 24–hour period to achieve rapid efficacy, comparable to the single-dose cure. The differential activity of CFZ between *Cryptosporidium* spp. raises an additional consideration that will require testing to determine the dose and regimen to treat *C*. *hominis*. These parameters will continue to be relevant as CFZ is evaluated in cryptosporidiosis clinical trials, and as the remaining 11 hits from this screen are investigated for further preclinical development.

## Materials and methods

### Ethics statement

This study was carried out in strict accordance with the recommendations in the Guide for the Care and Use of Laboratory Animals of the National Institutes of Health. The protocol (S13013) was approved by the Institutional Animal Care and Use Committee of the University of California, San Diego (Animal Welfare Assurance Number: A3033-01). All efforts were made to minimize suffering of animals employed in this study.

### Experimental compounds

Two compound libraries were assayed against *Cryptosporidium parvum* in vitro. The first, the Bioactives Set, was a compilation of 10,453 known bioactive compounds, including approved drugs and annotated tool compounds. The second was a diversity set designed by the University of Dundee Drug Discovery Unit, generously gifted for screening against *C*. *parvum*, totaling 68,689 unique drug-like compounds. The Global Health Chemical Diversity Library is a lead-like diversity library prepared for screening against the Bill and Melinda Gates Foundation priority pathogens. The compounds were commercially sourced from reputable vendors, selected to have lead-like physicochemical properties, enhanced Fsp3 character, enrichment in novel chemotypes, and filtered to remove unwanted groups. 200 compounds from this library were not tested due to insufficient source volume or transfer failures. Data from the GHCDL portion of the screen will be made available online from the University of Dundee Drug Discovery Unit, published at ChEMBL-Neglected Tropical Disease (https://www.ebi.ac.uk/chemblntd). Resupplied compounds for confirmation were high-purity powders (> 95% purity) from Selleck Chemicals, Calbiochem, Ambinter, Enzo Life Sciences, NCI, ChemDiv, or Life Chemicals. Screen control compounds were 30 μM nitazoxanide (Sigma-Aldrich) and 0.5 μM floxuridine (Sigma-Aldrich) as controls for *C*. *parvum* inhibition, and 10 μM puromycin (Sigma-Aldrich) for host cell cytotoxicity. Clofazimine (USP reference standard) was purchased from Sigma-Aldrich. All source plates were 384-well acoustic transfer-compatible plates (Labcyte) with compounds pre-diluted in dimethyl sulfoxide (DMSO) at either 1 mM (Bioactives) or 2 mM (all others). For single-point testing, compounds were transferred into 1536-well tissue culture-treated, black-walled, clear-bottomed, low base microwell plates (Greiner) with an ECHO liquid handler (Labcyte) to a final concentration of 1.88 μM. For dose-response testing, compounds were serially diluted 1:3 in 11-points and then transferred into triplicate 1536-well plates, with a top concentration of 12.5 μM. Primary screening was done with a single replicate, whereas single-point reconfirmation and dose-response testing was carried out in triplicate (3 technical replicates). The number of biological replicates for filtered hits is indicated in S2 Table.

### HCT-8 host cell culture

Human ileocecal adenocarcinoma (HCT-8; ATCC CCL244) cells were maintained in T-75 tissue culture flasks with RPMI 1640 medium supplemented with L-glutamine, 10% heat-inactivated fetal bovine serum (HI-FBS), 100 IU penicillin, and 100 mg/mL streptomycin. At confluency, cells were trypsinized, washed, and resuspended in assay medium: RPMI 1640 (-) phenol red, supplemented with 2% heat-inactivated horse serum (ATCC), 100 IU penicillin, and 100 mg/mL streptomycin. Cells were then plated (5 μL/well) into 1536-well assay plates at a density of 5.5×10^5^ cells/mL (2,750 cells/well). Cells were allowed to grow for 24 h at 37°C with 5% CO_2_ in a humidified tissue culture incubator.

### *Cryptosporidium* infection of HCT-8 cells

*C*. *parvum* oocysts (Iowa strain, isolated from infected calves) were purchased from the Sterling Parasitology Laboratory, University of Arizona, and stored at 4°C for ≤ 3 months in an antibiotic solution (0.01% Tween 20 containing 100 U/mL penicillin and 100 μg/mL gentamicin). *C*. *hominis* oocysts (TU502, isolated from gnotobiotic piglets) were purchased from the Tzipori Laboratory, Cummings School of Veterinary Medicine, Tufts University, and stored at 4°C for ≤ 2 weeks in an antibiotic solution (100 U/mL penicillin and 100 μg/mL streptomycin). For infection of assay plates, 24 h after cell seeding, oocysts were excysted and prepared for inoculation as previously described [49]. Briefly, the oocysts were diluted into 1 mL pre-warmed 10 mM HCl and then incubated in a 37°C water bath for 10 min. Oocysts were then pelleted at 3,000×*g* for 1 min in a microcentrifuge (Thermo), and the supernatant was carefully aspirated. Oocysts were resuspended in 2 mM sodium taurocholate in DPBS++ (with 0.9 mM CaCl_2_ and 0.5 mM MgCl_2_) and incubated at room temperature for 20 min. After incubation in bile salts, the oocysts were diluted 1:100 and free sporozoites and intact oocysts were enumerated to determine the percentage of excystation. The oocysts were then diluted with assay medium to 1.04×10^6^ oocysts/mL (3,125 oocysts/well) and dispensed (3 μL/well, 8 μL/well final volume) with a MultiFlo FX Multi-Mode Dispenser (Biotek). Plates were then spun at 150×*g* for 3 min in a Sorvall Legend XTR benchtop centrifuge (Thermo). Infected cells were incubated at 37°C with 5% CO_2_ in a humidified tissue culture incubator covered with metal assay lids (The Genomics Institute of the Novartis Research Foundation) for 48 h.

### High-content imaging of *Cryptosporidium* proliferation

Following incubation, infected cells were fixed with 4% paraformaldehyde for 15 min at room temperature. Plates were then washed twice with phosphate buffered saline (PBS). Prior to staining, cells were permeabilized with 0.25% Triton X-100 in PBS for a maximum of 15 min at room temperature, and then washed twice with PBS-T (1× PBS with 0.1% Tween 20). To prevent non-specific binding, the cells were blocked with SuperBlock^™^ T20 blocking agent (Thermo) for 1 h at room temperature. *Cryptosporidium* parasites were stained with 1 μg/mL fluorescein isothiocyanate (FITC)-conjugated *Vicia villosa* lectin (Vector Laboratories) in 1:10 diluted SuperBlock^™^ in PBS-T, supplemented with 3 μM 4',6-diamidino-2-phenylindole (DAPI) to visualize host cell nuclei. Staining was for 1 h at room temperature in the dark. Finally, cells were washed twice with PBS-T and the plates were sealed with adhesive foil. The plates were imaged with a CellInsight CX5 High Content Screening Platform (Thermo) with a 10× objective. Two channels were used: 384/440 nm for DAPI-stained nuclei, and 485/521 nm for FITC-lectin-labeled *Cryptosporidium* parasites. For 1536-well format assays (primary screening, triplicate reconfirmation, and dose-response reconfirmation, one microscopic field (802,511.39 μm^2^) per well, and for the 384-well washout assay, 4 fields per well were captured. The software identified primary objects (HCT-8 host cells) and spots within allowed distances to the nuclei (*Cryptosporidium*). Both cytotoxicity against HCT-8 cells (number of nuclei relative to DMSO-treated controls) and *Cryptosporidium* inhibition (spot counts relative to DMSO-treated controls) were assessed.

### Data analysis

Images were processed by the HCS Studio Scan software, and Selected Object Count (HCT-8 cells) and Spot Count (*Cryptosporidium*) were analyzed in Genedata Screener (v13.0-Standard). Spot Count and Selected Object Count were normalized to neutral controls minus inhibitors (floxuridine for Spot Count, and puromycin for Selected Object Count). For single-point primary assay plates, an additional run-wise median correction was applied to reduce screen artifacts (e.g. uneven dispense), whereas no correction was applied to triplicate reconfirmation and dose-response plates. For primary screening, a compound is considered a hit by a ≥ 70% reduction in Corrected Spot Count. Compounds that caused moderate cytotoxicity were filtered out (i.e., excluding all Corrected Selected Object Counts ≤ -60%). Dose-response curves were fit with Genedata Analyzer using the Smart Fit function. Final filtered hits included those with an EC_50_ (half-maximal effective concentration) ≤ 1 μM, with a CC_50_ (half-maximal cytotoxic concentration) ≥ 10-fold greater than the EC_50_ value.

### Mammalian cell cytotoxicity assays

Two mammalian cell lines were used for counter-screening for general cytotoxicity of hit compounds: human embryonic kidney cells (HEK293T; ATCC CRL-3216) and human hepatocellular carcinoma cells (HepG2; ATCC HB-8065). Each were maintained in T-150 tissue culture flasks with DMEM supplemented with 10% HI-FBS, 100 IU penicillin, and 100 mg/mL streptomycin. At 80% confluency, cells were trypsinized, washed, and resuspended in assay medium: DMEM supplemented with 2% HI-FBS, 100 IU penicillin, and 100 mg/mL streptomycin. Compounds were pre-spotted into tissue culture-treated white solid-bottomed 1536-well plates (Greiner) in a 1:3 dose-response dilution (top concentration 20 μM). HEK293T and HepG2 cells were diluted to 75×10^3^ cells/mL and 150×10^3^ cells/mL, respectively, and 5 μL/well were dispensed into assay plates with a MultiFlo FX Multi-Mode Dispenser (Biotek). Cells were incubated with metal lids (The Genomics Institute of the Novartis Research Foundation) at 37°C with 5% CO_2_ in a humidified tissue culture incubator for 72 h. At the completion of the assay, CellTiter-Glo (Promega) was prepared at 1:2 (reagent:water) of the manufacturer’s instructions, and 2 μL were dispensed into each well. After a 5 min incubation at room temperature, luminescence readings were measured with an EnVision Multilabel Plate Reader (Perkin Elmer). Relative fluorescence units were uploaded into Genedata Screener (v13.0-Standard), and data normalized to DMSO- and puromycin-treated wells. A four-parameter non-linear regression curve fit was applied to dose-response data using Genedata to determine the half maximal cytotoxic concentration (CC_50_) of each compound.

### Washout assay

Infected cells were treated in six 3-h increments over the course of 15 h (the presumed in vitro asexual lifecycle of *C*. *parvum*). HCT-8 cells were seeded (25 μL/well) into clear-bottomed 384-well plates (Greiner) at a density of 6.0×10^5^ cells/mL (15,000 cells/well) in assay medium, and allowed to grow 24 h at 37°C with 5% CO_2_ in a humidified tissue culture incubator, covered with a custom metal lid (The Genomics Institute of the Novartis Research Foundation) to reduce evaporation. After 24 h, and 2 h before infecting with *C*. *parvum* oocysts (time -2 hpi), the treatment time course was initiated. Compounds (NTZ, FDU, BKI-1294, and CFZ) were used at their EC_50_, EC_99_, and 3×EC_99_ value to determine the extent of parasite proliferation inhibition resulting from a 3-h exposure to each compound. DMSO was used as a negative control. Cell medium was removed from designated wells and replaced with medium containing either compound or DMSO. At time 0 (infection), *C*. *parvum* (Iowa strain) oocysts were excysted as above, diluted in assay medium to 1.04×10^6^ oocysts/mL (15,625 oocysts/well) and dispensed (15 μL/well, 40 μL/well final volume). Plates were then spun at 150×*g* for 3 min, and kept at 37°C with 5% CO_2_ in a humidified tissue culture incubator during drug treatment intervals. At the end of each 3 h time point, infected cells were carefully washed three times with fresh, pre-warmed assay medium, and the next set of wells for the subsequent time point were treated. The final time point extended to 18 h, to cover re-invasion of merozoites into new HCT-8 cells. After the final compound treatment was washed off, infected cells were allowed to grow until 48 h post infection.

To examine if cellular accumulation of compound was inhibiting parasite proliferation, host cells were pretreated for 3 h with NTZ, FDU, BKI-1294, or CFZ (in 1:3 11pt dose-response; top concentrations were 30 μM for NTZ; 0.5 μM for FDU; 12.5 μM for BKI-1294 and CFZ), washed, and then infected with *C*. *parvum*. A set of parallel wells were also treated and not washed prior to infection. 48 hpi, infected cells were imaged as described above, and EC_50_s for each compound pre- and post-wash were determined.

### Pharmacokinetic studies of clofazimine in mice

Male CD-1 fasted mice (three per group) were dosed per os with 20 mg/kg CFZ, formulated at 4 mg/mL in either 100% corn oil or 0.5% methylcellulose/0.5% Tween 80, or 20 mg/kg BKI-1294 formulated at 4 mg/mL in 7% Tween 80, 3% ethanol, and 90% water, and then monitored for plasma concentration, renal excretion, fecal excretion, as well as other clinical markers of adverse events (severe weight loss, lethargy, hunched posture, social isolation) for a total of 72 h.

### Mouse cryptosporidiosis model

Four week old female C57BL/6 IFNγ^-/-^ mice were purchased from the Jackson Laboratory and acclimated for four to ten days in specific pathogen-free conditions in the Health Sciences Biomedical Research Facility at the University of California, San Diego. Mice were provided water and chow (Teklad 2920X) ad libitum. For inoculation, *C*. *parvum* oocysts were adjusted to a final density of 5×10^4^/mL in cold, sterile, distilled water. Mice were infected via oral gavage with 200 μL (10^4^ oocysts) using a 20G×1.5” feeding needle. On days 4, 5, and 6 post-infection, mice were gavaged with 10 mL/kg clofazimine (USP reference standard, Sigma Aldrich) solubilized in food-grade corn oil. Treatment regimens included 10 mg/kg, 100 mg/kg, and 0 mg/kg (vehicle only; n = 4 per group). Certain assays also involved gavage of characterized anticryptosporidial BKI-1294 (a kind gift from W. Van Voorhis, U Washington), administered at 10 mg/kg mouse as a 10 mL/kg emulsified suspension in 0.5% methylcellulose + 0.5% Tween-80. At intervals between 3 and 30 days post-infection mice were weighed and temporarily placed in isolation to allow for collection of feces (3 pellets/mouse/sampling point). Pellets were weighed and placed in 0.5 mL 2.5% potassium dichromate solution, and stored at 4°C until processing.

### Quantitative detection of fecal oocyst load

Oocysts were extracted via a modified discontinuous sucrose gradient technique [50]. Briefly, Sheather’s solution was freshly prepared by dissolving 156.25 g sucrose and 2.5 mL phenol in 100 mL water. Fecal pellets were removed from storage then homogenized by vortexing and pipetting. In microcentrifuge tubes, 0.2 mL fecal homogenate was overlaid on 0.75 mL of solution with a specific gravity of 1.064 (20% Sheather’s and 0.1% Tween 80 in PBS), overlaid on 0.75 mL of solution with a specific gravity of 1.103 (33% Sheather’s and 0.1% Tween 80 in PBS). Oocysts were floated from feces by centrifugation at 1,000×*g* for 20 min and collected with a pipet tip from the 1.064/1.103 specific gravity interface. Oocysts were rinsed once in cold PBS, pelleted by centrifugation at 15,000×*g* for 10 min, and resuspended in PBS. 50 μL aliquots were incubated for 30 min at room temperature with 0.25 μg fluorescein isothiocyanate-conjugated mouse anti-*Cryptosporidium* antibody (OW50-FITC, BioRad 2402–3007), then diluted to 200 μL with PBS. Samples were analyzed using a Guava EasyCyte flow cytometer and CytoSoft Data Acquisition and Analysis software (v5.3; Guava Technologies, Inc.), using a 100 s sampling interval, 0.59 μL/s flow rate, and logical gating of forward and side light scatter and OW50-FITC fluorescence signals. Each experimental run included positive and negative controls to calibrate region settings discriminating the oocyst-FITC population from background signals. Oocyst counts/mL sample values were exported to Excel (Microsoft Corp.) for normalization to counts/mg feces. Final graphing and statistical analyses of data were done using GraphPad Prism software (v6, GraphPad Software, Inc.). Two-way ANOVA with Dunnett’s correction (Fig 4A) and unpaired multiple t-tests with False Discovery Rate approach (Fig 4B) were performed on % Vehicle data to determine significance between vehicle-treated and compound-treated mice.

## Supporting information

S1 Table Pilot screening statistics. (PDF)

S2 Table Activities of 12 filtered hits against *Cryptosporidium* spp., host cells, and mammalian cytotoxicity lines. Screening controls, BKI-1294, and 12 filtered hits from both libraries. Compounds from Bioactives and GHCDL are sorted by potency against *C*. *parvum*.
(XLSX)

S3 Table Accumulation of clofazimine from pre-treatment of host cells inhibits *C*. *parvum* growth. Both floxuridine and clofazimine exhibit activity even after washout, indicating that an adequate amount of compound accumulated in host-cells to affect parasite proliferation within the 48 h assay timeframe.
(PDF)

S1 Fig Structures of top screening hits. The five most potent compounds against *C*. *parvum* were from the Bioactive library: Gö6976 (EC_50_ = 2.5 nM), monensin (EC_50_ = 7.0 nM), clofazimine (EC_50_ = 15 nM; Fig 4A), cyclosporine (EC_50_ = 48 nM), and MST-312 (EC_50_ = 61 nM). GHCDL-1 (EC_50_ = 1.42 μM) and GHCDL-2 (EC_50_ = 293 nM) were the only two hits that reconfirmed from the GHCDL with an EC_50_ ≤ 1 μM and CC_50_/EC_50_ ≥ 10-fold upon initial reconfirmation testing.
(TIFF)

S2 Fig Washout data of clofazimine and controls at their EC_50_ and 3×EC_99_. The first asexual life cycle after infection was evenly divided into six 3-h blocks, and labeled as hours post infection (hpi). Infected cells were treated by one of four compounds at either the EC_50_ (A) or 3×EC_99_ (B) for 3 h followed by drug washout, and then allowed to continue growing until 48 hpi, when they were fixed, stained, imaged, and analyzed for *C*. *parvum* proliferation. EC_50_, 3×EC_99_ values: NTZ = 2.8 μM, 24 μM; FDU = 17 nM, 300 nM; CFZ = 15 nM, 90 nM; BKI-1294 = 400 nM, 6 μM. Data shown are the mean ± SEM of two independent experiments.
(TIFF)

S3 Fig Percent recovery of clofazimine and BKI-1294 in urine. Unchanged CFZ formulated in either corn oil (solution) or MC-Tween (suspension), or BKI-1294 recovered in the urine of mice dosed in Fig 5. Recovery was measured each day for three days. Data shown are mean ± SEM (n = 3).
(TIFF)
