# Supplementary material for: A high-throughput phenotypic screen identifies clofazimine as a potential treatment for cryptosporidiosis
Source: PLoS Negl Trop Dis. 2017 Feb 3;11(2):e0005373. doi: 10.1371/journal.pntd.0005373 (PMC5310922; doi:10.1371/journal.pntd.0005373)
Supplement: S1 Table — (PDF) [file pntd.0005373.s001.pdf]

**Statistic**	**Value**
Average Spot Count – DMSO	829
Average Spot Count – FDU	143
Standard Deviation – DMSO	183
Standard Deviation – FDU	55
%CV – DMSO	22.0
%CV – FDU	38.6
Window	5.81
Z’ Factor[a]	0.24
Robust Z’	0.45

[a]Z’ Factor is defined as: $Z' = 1 - \frac{3(\sigma_p + \sigma_n)}{|\mu_p - \mu_n|}$
