# Supplementary material for: A high-throughput phenotypic screen identifies clofazimine as a potential treatment for cryptosporidiosis
Source: PLoS Negl Trop Dis. 2017 Feb 3;11(2):e0005373. doi: 10.1371/journal.pntd.0005373 (PMC5310922; doi:10.1371/journal.pntd.0005373)
Supplement: S3 Table — Both floxuridine and clofazimine exhibit activity even after washout, indicating that an adequate amount of compound accumulated in host-cells to affect parasite proliferation within the 48 h assay timeframe. (PDF) [file pntd.0005373.s003.pdf]

**Compound**	**Pre-wash EC_{50} (µM)**	**Post-wash EC_{50} (µM)**
Nitazoxanide	>30	2.53
Floxuridine	0.053	0.0094
BKI-1294	>12.5	0.955
Clofazimine	0.023	0.0070
