# Supplementary figures and images for: A high-throughput phenotypic screen identifies clofazimine as a potential treatment for cryptosporidiosis
Source: PLoS Negl Trop Dis. 2017 Feb 3;11(2):e0005373. doi: 10.1371/journal.pntd.0005373 (PMC5310922; doi:10.1371/journal.pntd.0005373)

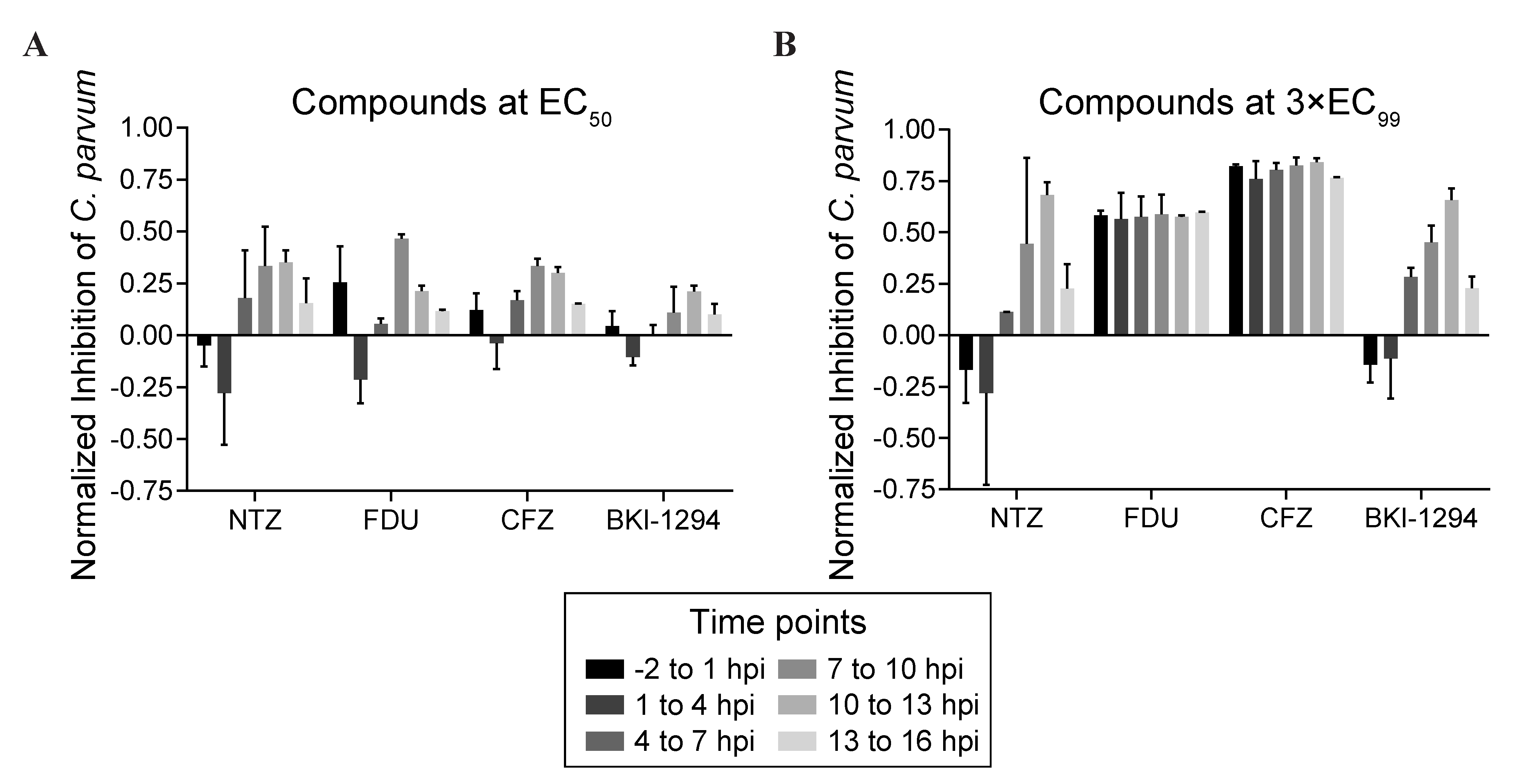

Supplement: S2 Fig — The first asexual life cycle after infection was evenly divided into six 3-h blocks, and labeled as hours post infection (hpi). Infected cells were treated by one of four compounds at either the EC50 (A) or 3×EC99 (B) for 3 h followed by drug washout, and then allowed to continue growing until 48 hpi, when they were fixed, stained, imaged, and analyzed for C. parvum proliferation. EC50, 3×EC99 values: NTZ = 2.8 μM, 24 μM; FDU = 17 nM, 300 nM; CFZ = 15 nM, 90 nM; BKI-1294 = 400 nM, 6 μM. Data shown are the mean ± SEM of two independent experiments. (TIFF) [file pntd.0005373.s005.tiff]

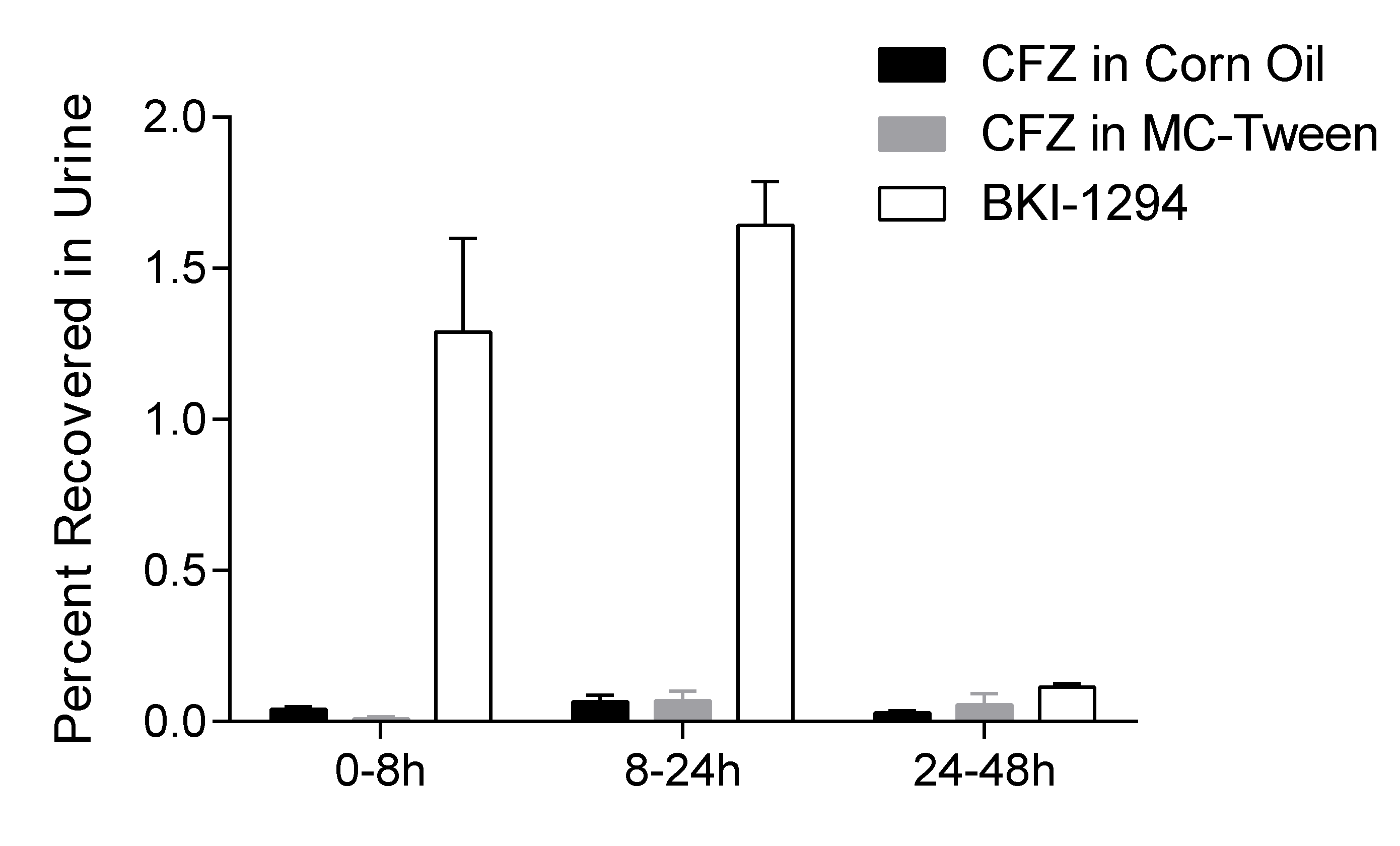

Supplement: S3 Fig — Unchanged CFZ formulated in either corn oil (solution) or MC-Tween (suspension), or BKI-1294 recovered in the urine of mice dosed in Fig 5. Recovery was measured each day for three days. Data shown are mean ± SEM (n = 3). (TIFF) [file pntd.0005373.s006.tiff]
